# Identification of oogonial stem cells in chicken ovary

**DOI:** 10.1111/cpr.13371

**Published:** 2022-12-16

**Authors:** Lu Meng, Yun Zhang, Yao Hua, Yuxiao Ma, Heng Wang, Xianyao Li, Yunliang Jiang, Guiyu Zhu

**Affiliations:** ^1^ Shandong Provincial Key Laboratory of Animal Biotechnology and Disease Control and Prevention, College of Animal Science and Veterinary Medicine Shandong Agricultural University Taian China; ^2^ College of Animal Science and Technology Huazhong Agricultural University Wuhan China

## Abstract

**Objectives:**

Oogonial stem cells (OSCs) are germ cells that can sustain neo‐oogenesis to replenish the pool of primary follicles in adult ovaries. In lower vertebrates, fresh oocytes are produced by numerous OSCs through mitosis and meiosis during each reproduction cycle, but the OSCs in adult mammals are rare. The birds have retained many conserved features and developed unique features of ovarian physiology during evolution, and the presence of OSCs within avian species remain unknown.

**Materials and Methods:**

In this study, we investigated the existence and function of OSCs in adult chickens. The chicken OSCs were isolated and expanded in culture. We then used cell transplantation system to evaluate their potential for migration and differentiation in vivo.

**Results:**

DDX4/SSEA1‐positive OSCs were identified in both the cortex and medulla of the adult chicken ovary. These putative OSCs undergo meiosis in the reproductively active ovary. Furthermore, the isolated OSCs were expanded in vitro for months and found to express germline markers similar to those of primordial germ cells. When transplanted into the bloodstream of recipient embryos, these OSCs efficiently migrated into developing gonads, initiated meiosis, and then derived oocytes in postnatal ovaries.

**Conclusions:**

This study has confirmed the presence of functional OSCs in birds for the first time. The identification of chicken OSCs has great potential for improving egg laying and preserving endangered species.

## Graphical abstract

The oogonial stem cells (OSCs) could support the formation of new oocytes in the adult ovary. The DDX4/SSEA1‐positive OSCs in chicken ovaries could be isolated and expanded in vitro while maintaining germline stemness. When transplanted into the developing ovaries, the chicken OSCs can proliferate and enter meiosis to produce oocytes. The chicken OSCs could offer new ways for improving egg laying performance and species conservation.

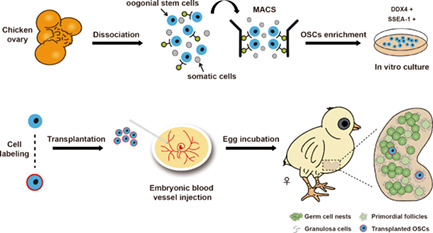

## 1 INTRODUCTION

In adult ovaries, the oogonial stem cells (OSCs) have the regenerative capability to produce new oocytes and contribute to the replenishment of primary follicles. Mammalian OSCs have attracted tremendous interest among reproductive biologists and clinicians because of their great potential in the treatment of infertility. It was traditionally believed that the mammalian ovary has a finite number of follicles at birth and becomes depleted over time, since the germ cells enter meiosis and cease division after birth.^1^ Scientists have also long considered that adult mouse ovaries neither acquire OSCs nor produce new oocytes in vivo. Instead, the finite primordial follicle pool is the only source of follicle generation for regular ovulation.^2^

However, the traditional dogma of “no oocyte generation after birth in mammals” was challenged by several recent reports, which showed the existence of mitotically active female germline progenitors in mammalian postnatal ovaries, particularly in mouse models.^2^, ^3^, ^4^ For instance, the presence of Bromodeoxyuridine (BrdU)/MVH (mouse vasa homologue) double‐positive cells in ovarian surface epithelium (OSE) provides strong evidence for germ‐cell proliferation and follicle renewal in the postnatal mouse ovary.^3^ Subsequently, the presence of pluripotent stem cell‐like cells within the OSE has been identified in rats,^5^ pigs,^6^ sheep,^7^ cows,^8^ monkeys^9^ and even biopsies of menopausal human tissue,^10^ and various degrees of germline potential have been demonstrated in these cells. In rodents, the OSCs have been isolated, cultured and differentiated into oocytes after transplantation back into the ovaries of females.^11^ Although extremely rare, the OSCs in the adult human ovarian cortex have been successfully isolated^2^, ^12^ and verified with the sensitive single‐cell RNA‐seq.^13^ Nevertheless, how to efficiently mobilize and utilize this rare OSC population in adult mammals remains challenging.

There is no doubt that plenty of OSCs exists in the adult ovaries of many non‐mammalian vertebrates, including fish,^14^ amphibians,^15^ and reptiles.^16^ OSCs in lower vertebrates maintain high proliferative capacity and support the continuous production of oocytes during postnatal life.^14^ However, the existence of OSCs in birds has not yet been investigated. The reproductive biology of birds is dramatically different from that of other vertebrates, and avian female germ cells also exhibit many unique features throughout their lifespan. In chickens, the oogonia are derived from primordial germ cells (PGCs), which first appear in the central region of the blastoderm. After migration into the germinal crescent, the chicken PGCs then penetrate the blood vessels and travel long distances to reach the genital ridge and arrive at the embryonic gonads.^17^, ^18^ Then, the female germ cells begin to proliferate significantly at embryonic day 9 (E9), initiate massive meiosis in response to retinoic acid signalling starting from E15.5, and then arrest at prophase I.^19^, ^20^ These germ cells give rise to the oocytes and establish the pool of primordial follicles, which serve as the only source for gamete production in the ovary. It remains unclear whether most, rather than all, female germ cells enter meiosis during the embryonic meiotic wave and whether any escaped germ cells could develop into adult OSCs afterwards.

In this study, we provide the first line of evidence suggesting that OSCs do exist in young and adult laying chicken ovaries. The isolated OSCs have characteristics similar to those of PGCs and embryonic stem cells. When reintroduced into embryonic blood vessels, these cells are capable of gonadal colonization and subsequent meiosis initiation to produce oocytes.

## 2 MATERIALS AND METHODS

### 2.1 Animals and tissue preparation

Fertilized eggs from Hy‐Line chicken were incubated in an incubator at 37.8°C and 60% humidity. The gonads were collected at E16.5 and E18.5. The ovarian tissues from early laying (Day 100) and peak laying (Day 200) Hy‐line chicken were collected from the local research farm of Huazhong Agricultural University. Guidelines for utilization of animals in research were followed according to the standards of Huazhong Agricultural University Animal Care and Use Committee.

### 2.2 Isolation of OSCs

The tissues were collected from different ovaries and washed five times in phosphate‐buffered saline (PBS) (Hyclone). Then the tissues were dissociated in PBS containing 1 mM EDTA and 0.25% trypsin (Sigma‐Aldrich) at 37°C for 15–20 min until most of the cells were dispersed. The trypsin was neutralized by adding 10% fetal bovine serum (Hyclone). The OSCs were enriched by immunomagnetic isolation using stage‐specific embryonic antigen‐1 (SSEA‐1) antibody (Santa Cruz, sc‐21,702) conjugated with anti‐mouse IgM Dynabeads (Invitrogen, 11039D).

### 2.3 Culture of OSCs

The OSCs were cultured in a 12‐well glass bottom plate (NEST Biotechnology) pre‐seeded with irradiated chicken embryo fibroblasts feeder. The culture medium is KnockOut‐Dulbecco's Modified Eagle Medium (KO‐DMEM) (Gibco) containing 6 ng/ml basic fibroblast growth factor (bFGF) (Solarbio), 25 ng/ml Human Activin A (Novoprotein), 7.5% defined fetal bovine serum (Hyclone), 2.5% chicken serum (Solarbio), 1 × antibiotic‐antimycotic (Gibco), 1 × GlutaMAX (Gibco), 1 × NEAA (Gibco), 1 × B‐27 Supplement (Gibco), 1 × EmbryoMax Nucleosides (Millipore), 1.2 mM Sodium Pyruvate (Gibco), and 0.1 mM β‐mercaptoethanol (Sigma‐Aldrich). Cells were cultured at 39°C in 5% CO_2_, with fresh medium replaced every other day, then they were subcultured every 3–5 days upon confluence. The PGCs were isolated from the E5.5 gonad according to a previous protocol^21^ and maintained with the same conditions of OSCs.

### 2.4 Isolation of granulosa cell and theca cell

The preovulatory follicles were collected and washed three times with PBS. The follicles were punctured to release the yolk and the granulosa layers were separated from the theca layers according to the method of the previous report.^22^ The granulosa layers were chopped with scissors and placed in PBS containing 1 mM EDTA and 0.25% trypsin (Sigma‐Aldrich) at 37°C for 7–8 min. The theca layers were digested for 20 min. When most of the cells were dispersed, trypsin was neutralized by adding 10% fetal bovine serum (Hyclone). Cells were collected by centrifugation and resuspended in the RPMI‐1640 medium containing 10% fetal bovine serum.

### 2.5 Trypan Blue staining

The cultured cells were centrifuged at 100 × g for 5 min and the cell pellet was collected. Then the OSCs and PGCs were resuspended in 1 ml PBS mixing with 0.4% trypan blue. Allow mixture to incubate 5 min and count the unstained (lived) and stained (dead) cells in the hemacytometer.

### 2.6 Immunofluorescence staining

The ovarian sections were permeabilized in 0.5% Triton X‐100 for 10 min and incubated in blocking solution (6% horse serum in PBS, 30 min, room temperature), followed by incubation with the primary antibodies in a blocking solution overnight at 4°C, and then secondary antibodies prepared in a blocking solution at room temperature for 60 min. Sources and dilution of primary antibodies were as follows: anti‐SSEA1 antibody (1:200, Santa Cruz, sc‐21,702), anti‐DDX4 antibody (1:200, Abcam, ab13840), anti‐phosphorylation of H2AX on serine 139 (γH2AX) antibody (1:200, Abcam, ab26350), anti‐synaptonemal complex protein 3 (SCP3) antibody (1:200, Fisher Scientific, NB30231A350), anti‐BMP15 antibody (1:100, Santa Cruz, sc‐271,824) and anti‐NOBOX antibody (1:100, Santa Cruz, sc‐514,178). Secondary antibodies were Alexa Fluor 488 goat anti‐mouse IgM (1:200, Invitrogen, A‐21042), Alexa Fluor 488 donkey anti‐rabbit Immunoglobulin G (IgG) (1:200, Invitrogen, A‐21206), Alexa Fluor 555 goat anti‐mouse Immunoglobulin M (IgM) (1:200, Solarbio, K0055G‐AF555), Alexa Fluor 555 goat anti‐mouse IgG (1:200, Invitrogen, A‐21127) and Alexa Fluor 555 donkey anti‐rabbit IgG (1:200, Invitrogen, A‐31572). Counterstaining of the nucleus was performed using 4′,6‐diamidino‐2‐phenylindole (DAPI) (Invitrogen).

The cultured cells were fixed with 4% paraformaldehyde for 10 min. After fixation, the cells were permeabilized in 0.5% Triton X‐100 for 10 min and incubated in blocking solution (6% horse serum in PBS, 30 min, room temperature), followed by incubation with the primary antibodies in a blocking solution overnight at 4°C with anti‐SSEA1 (1:200, Santa Cruz, sc‐21,702) and anti‐DDX4 (1:200, Abcam, ab13840). After washing in PBS, OSCs were incubated with Alexa Fluor 488 donkey anti‐rabbit IgG (1:200, Invitrogen, A‐21206) and Alexa Fluor 555 goat anti‐mouse IgM (1:200, Solarbio, K0055G‐AF555) secondary antibodies. Counterstaining of the nucleus was performed using DAPI (Invitrogen).

### 2.7 EdU Staining

To determine the proliferation of cultured OSCs, cells were incubated overnight in media supplemented with 10 μM EdU (Invitrogen, E10187). After formaldehyde fixation, cells were rinsed once with PBS and permeabilized in 0.5% Triton X‐100 for 10 min, then stained by incubating for 30 min with Tris (Invitrogen, AM9855G), CuSO4, fluorescent azide 555 (Invitrogen, A20012) and ascorbic acid (Sigma, A4403). After staining, the EdU‐stained cells can be immunostained by using conventional protocols as previously mentioned.^23^

### 2.8 RNA extraction and RT‐qPCR analysis

Total RNA was extracted with PicoPure RNA Isolation Kit (Thermofisher). In total, 1 μg of RNA was reverse transcribed using a PrimeScript RT reagent Kit with gDNA Eraser (TaKaRa). The qPCR was carried out in triplicate with a Hieff qPCR SYBR Green MIX (Yeasen). Quantitative reverse transcription PCR (RT‐qPCR) primers including SCP3, STRA8, SPO11, DMC1, DDX4, SSEA‐3, Oct4, Sox2, Nanog, C‐kit, C‐myc, Sall4, Prdm1, Dazl, AMH, Foxl2, Cyp19a1, Star, INSL3 and Cyp17a1 are listed in Table S1.

### 2.9 Cell transplantation

Chicken embryos incubated for 60 h were used as host for cell transplantation assay. The cultured OSCs and granulosa cells (GCs) were labelled with PKH26 (Sigma‐Aldrich), and a single‐cell suspension of 1.5 μl containing 1 × 10^4^ cells, was injected into bloodstream of embryos using a micro glass needle. Eggshells were sealed with paraffin wax, and embryos were transferred to an incubator. Gonadal or ovarian samples were collected at three time points, at E12.5 (9–10 days before hatching), E18.5 (3–4 days before hatching) and postnatal day 4 (4 days after hatching). The embryonic gonads were isolated and examined for cell migration under a stereo fluorescence microscope and then cryosectioned for immunofluorescence staining of meiosis markers. The 4‐day‐old host chickens were sacrificed and the left ovaries were isolated, fixed and sectioned for further immunofluorescence staining of oocyte markers as previously mentioned.

### 2.10 Statistical analysis

All data were expressed as mean ± SEM from 3 to 5 independent experiments. Student's *t‐*test was used to calculate the differences between experimental groups. Statistical analysis was performed by GraphPad Prism 8.0 software. Values were considered statistically significant at *p* < 0.05.

## 3 RESULTS

### 3.1 Identification of putative OSCs in chicken ovaries

**FIGURE 1:**
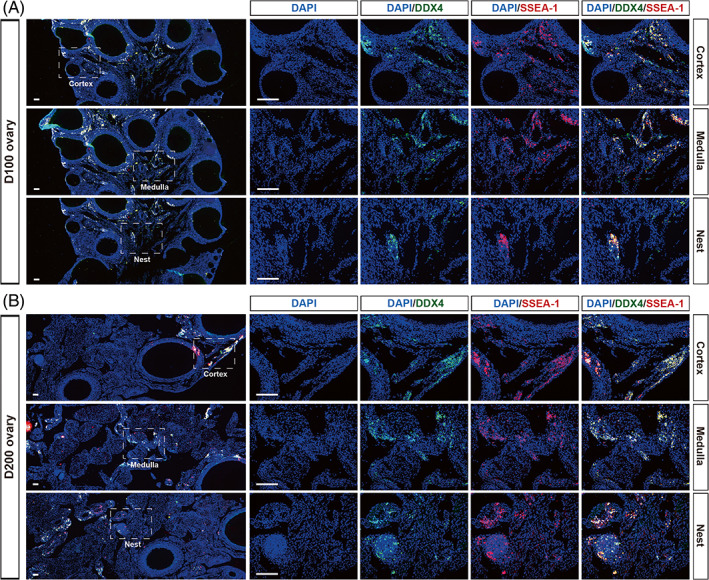
The identification of putative oogonial stem cells (OSCs) in adult chicken ovaries. (A) Ovary sections were immunostained for classical markers of OSCs. White boxes outline the areas demonstrating that some DDX4–SSEA1 double‐positive cells can be detected in the region of the cortex, medulla and nests. Dual immunofluorescence staining for DDX4 and SSEA‐1 in 100 days chicken ovaries. Scale bar = 100 μm. (B) Dual immunofluorescence staining for DDX4 and SSEA‐1 in 200 days chicken ovaries. Scale bar = 100 μm.

To verify the existence of OSCs in adult chickens, we collected ovaries from 100‐day‐old (D100) and 200‐day‐old (D200) chickens, ages that represent the early and peak laying periods, respectively. The presence of OSCs was examined by using the two classical and best‐recognized OSC markers: DDX4/VASA (DEAD‐box polypeptide 4), which is expressed exclusively in germ cells,^24^ and SSEA‐1, which is a pluripotent stem cell marker.^25^ Immunofluorescence staining revealed the existence of many DDX4‐SSEA1 double‐positive cells in the different layers of the ovary, including the cortex and medulla, of 100 days (Figure 1A) and 200 days (Figure 1B) adult chickens, indicating that OSCs might be present in the adult chicken ovary.

Previous studies suggested that germline stem cells mainly reside in the cortex of the ovary in mice,^3^ pigs,^6^ and humans.^26^ Comparably, our results showed that there were more OSCs in the ovarian cortex than in other regions in chicken. Additionally, we also found DDX4‐SSEA1 double‐positive cells located within ‘nests’ in the ovarian medulla, similar to the germ cell nests observed in other species,^27^ suggesting that a small percentage of OSCs were stored in the nests. Thus, our immunostaining confirmed the presence of putative OSCs in the active laying ovaries of chickens and showed that the majority of the OSCs were located in the cortex region.

### 3.2 OSCs are mitotically active in the ovaries of adult laying hens

**FIGURE 2:**
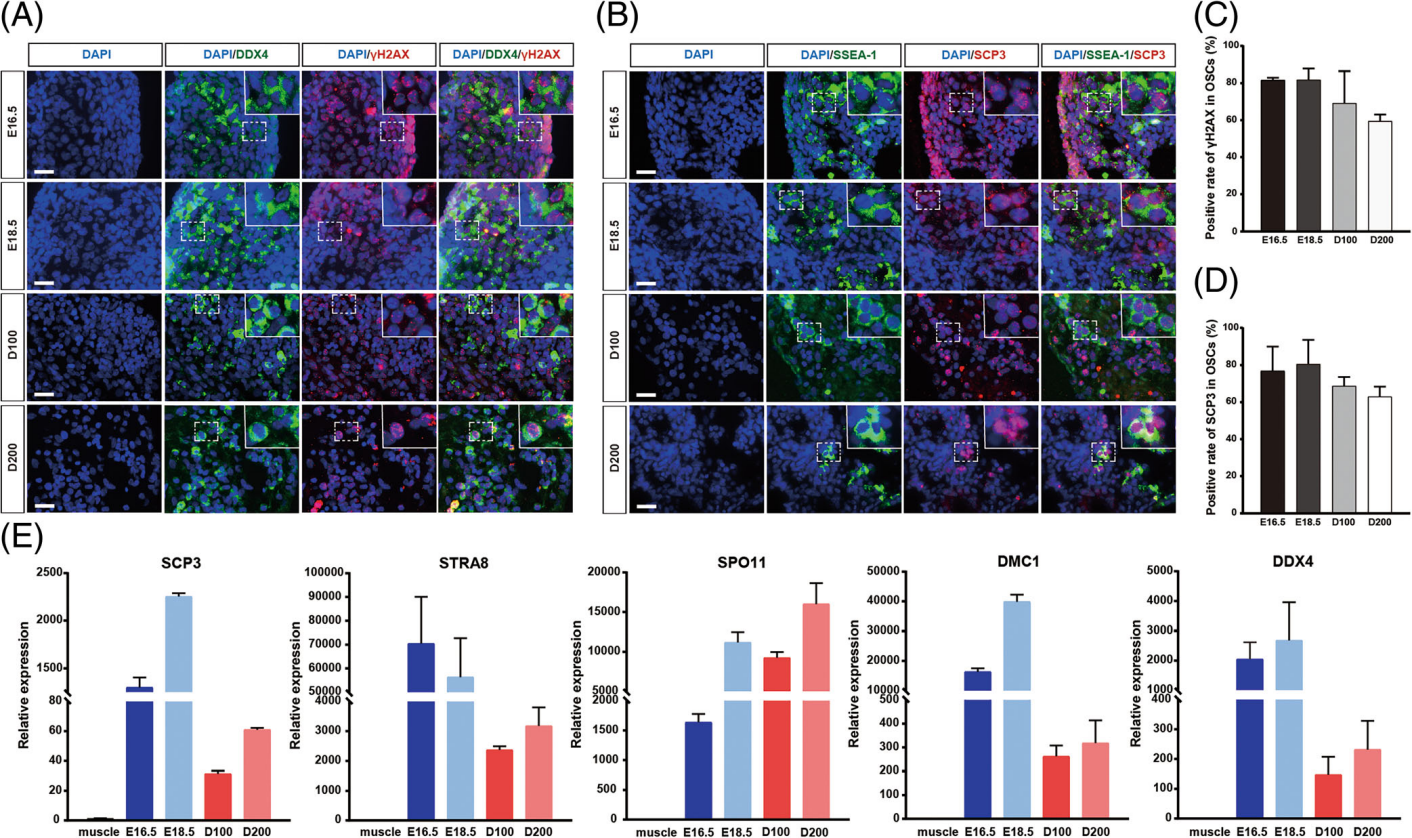
The OSCs undergo meiosis in chicken ovaries. (A) Representative images showing dual immunofluorescence for DDX4 and γH2AX in gonads and ovaries from different developmental stages, including embryonic day 16.5 (E16.5), embryonic day 18.5 (E18.5), postnatal day 100 (D100), postnatal day 200 (D200). The area indicated by the dashed box in the overview is magnified in the inset. Scale bar = 20 μm. (B) Representative images showing dual immunofluorescence for SSEA‐1 and SCP3 in gonads and ovaries from different developmental stages. The area indicated by the dashed box in the overview is magnified in the inset. Scale bar = 20 μm. (C) The proportion of meiotic OSCs as detected with DDX4 and γH2AX in embryonic gonads and adult ovaries. (D) The proportion of meiotic OSCs as detected with SSEA‐1 and SCP3 in embryonic gonads and adult ovaries. (E) The expression levels of marker genes of meiosis (SCP3, STRA8, SPO11 and DMC1) and germline (DDX4) in gonads and ovaries from different developmental stages. Muscle tissue was used as the negative control. The data are the mean ± SEM (*n* = 3).

In chickens, the onset of the major meiotic wave in female germ cells occurs around E15.5.^20^ Putative OSCs may escape the first meiotic wave during embryonic development, but whether they can enter meiosis later in adulthood is unclear. Thus, we utilized classical meiosis markers to search for meiotic OSCs in the ovary. The SCP3 and γH2AX are both specifically expressed as the earliest responses to signalling for initiating meiotic recombination and are involved in the processes of gametogenesis.^28^, ^29^ We first verified the identity of the meiotic female germ cells during embryonic development by coimmunostaining with the germ cell (DDX4, SSEA1) and meiotic markers (γH2AX, SCP3; Figure 2A,B). We found that the majority of the germ cells (>80%) were undergoing meiosis around E16.5 and E18.5, as shown by the high proportion of γH2AX‐ or SCP3‐positive signals among the germ cells in female embryos (Figure 2C,D). However, the initiation of meiosis in embryonic female germ cells is thought to be asynchronous. We proposed that a few germ cells would bypass embryonic meiosis to generate OSCs. Thus, we continued to examine the meiotic ability of these cells in adult ovaries. We observed γH2AX‐DDX4 and SCP3‐SSEA1 double‐positive cells in the cortex in reproductively active ovaries, although the proportions of meiotic germ cells were lower than those in the embryos (Figure 2C,D). Correspondingly, the RT‐qPCR analysis of the meiotic marker genes, including SCP3, STRA8, SPO11 and DMC1, indicated much higher levels in the embryonic gonads than in the adult ovaries (Figure 2E). Therefore, adult OSCs could enter meiosis, but the meiotic activity of germ cells declined with age. These findings were in agreement with a previous report in mice.^3^

### 3.3 The maintenance of germline potential and expression of pluripotency markers in cultured OSCs

**FIGURE 3:**
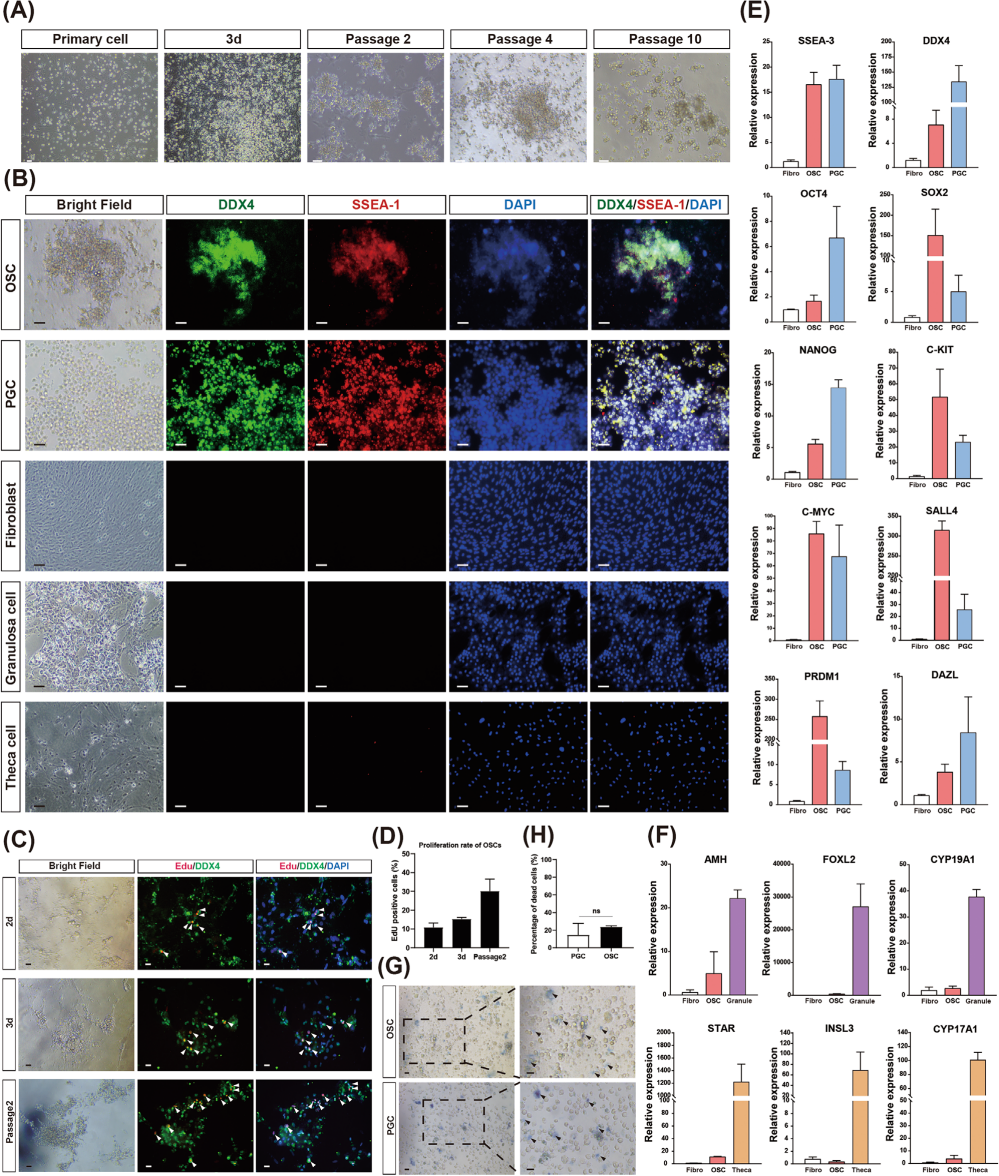
Chicken oogonial stem cells (OSCs) maintain germline characters after long‐term culture. (A) The cellular morphology of cultured OSCs isolated from chicken ovaries. (B) Representative images showing immunofluorescence staining for DDX4 and SSEA‐1 in cultured OSCs and PGCs. Chicken embryonic fibroblast cells (CEFs), granulosa cells (GCs) and theca cells (TCs) were used as negative controls. (C) Representative images of EdU staining and immunostaining for DDX4 in cultured OSCs. The arrowheads indicate EdU‐DDX4 double‐positive cells. (D) Statistical analysis of the proportion of EdU‐positive proliferative OSCs. (E) The expression levels of pluripotent stem cell markers (SSEA‐3, Sox2, Nanog, C‐myc) and germ cell markers (Oct4, DDX4, Prdm1, Dazl, C‐kit, Sall4) in OSCs, PGCs and fibroblasts. (F) The expression levels of granulosa cell markers (AMH, Foxl2, Cyp19a1) and theca cell markers (Star, INSL3, Cyp17a1) in OSCs, GCs and TCs. (G) Trypan blue staining showed similar cell viability in OSCs and PGCs after freezing and thawing. Arrowheads indicate stained (dead) cells. (H) The percentage of dead cells in OSCs and PGCs after freezing and thawing. Data are the mean ± SEM (*n* = 5), ns, not significant by Student's *t*‐test. Scale bar = 20 μm.

Next, we tried to isolate the putative OSCs from chicken ovaries with the magnetic‐assisted cell sorting (MACS) by using the DDX4 and SSEA‐1 antibodies. The current DDX4‐based MACS did not yield enough authentic cells for further functional analysis, possibly due to the lack of species cross‐reactivity of the DDX4 antibody. Nevertheless, the SSEA‐1, which is the most common marker protein used for chicken PGC isolation,^30^, ^31^ could give optimal and stringent cell output. Therefore, the OSCs of adult ovaries were isolated by SSEA1‐based magnetic bead sorting and maintained in culture conditions identical to those of PGCs.^32^ Immunostaining demonstrated that SSEA‐1 positive cells were almost 100% overlapped with DDX4 signal (Figure S1), confirming the identity and purity of chicken OSCs. The OSCs could grow together in aggregates and form spherical colonies comprising compact clusters of small round cells in a few days (Figure 3A). The majority of cultured OSCs (>90%) retained germ cell characteristics after multiple passages for 2 months, as shown by SSEA‐1 and DDX4 protein expression (Figure 3B). In comparison, the PGCs showed homogenous SSEA‐1 and DDX4 immunostaining signals (Figure 3B). Hence, similar to the PGCs, the chicken OSCs could also maintain the germline features after long‐term in vitro culture.

The cell proliferation in cultured OSCs was further examined by EdU assay. At Day 2, EdU‐DDX4 double‐positive cells were found in scattered individual cells. After continuous passage, EdU incorporation was predominately observed in the cell clusters, indicating the OSCs were actively proliferating and forming cell colonies (Figure 3C,D). Furthermore, we used RT‐qPCR to examine the expression of representative marker genes for pluripotency (SSEA‐3, Sox2, Nanog and C‐myc), germline (Oct4, DDX4, Prdm1, Dazl, C‐kit and Sall4), and follicular somatic cells (AMH, Foxl2, Cyp19a1, Star, INSL3 and Cyp17a1). The results showed that both the cultured OSCs and PGCs expressed high levels of stem cell and germline genes but not somatic genes (Figure 3E,F). In other species, a small number of ovarian somatic cells also possess germline competency and are capable of producing oocytes under various induction conditions.^33^ Thus, we examined germline gene expression in other sources of potential stem cells derived from the chicken ovary. We found no evidence of germline gene expression in ovarian somatic cells, such as GCs, TCs and fibroblast cells (Figure 3B). Accordingly, the marker genes for GCs (AMH, Foxl2 and Cyp19a1) and TCs (Star, INSL3 and Cyp17a1) were almost undetectable in cultured OSCs (Figure 3F). Hence, we could exclude the possibility of somatic cell origin of the chicken OSCs and confirm that they were derived from embryonic germ cells.

Freezing is the most effective method of maintaining a stable supply of various cell types for long‐term storage. Therefore, we examined whether the OSCs could withstand cryopreservation. As shown by trypan blue staining, there were no differences in the cell viability of OSCs and PGCs after freeze–thaw cycles (Figure 3G,H). Therefore, the OSCs collected from the adult ovaries could maintain their germ cell properties during long‐term culture in vitro, and the recovery rate and viability of OSCs after freezing and thawing were similar to those of PGCs. We next continued to test their germline competency in vivo.

### 3.4 The migration and differentiation of OSCs in chicken ovaries

**FIGURE 4:**
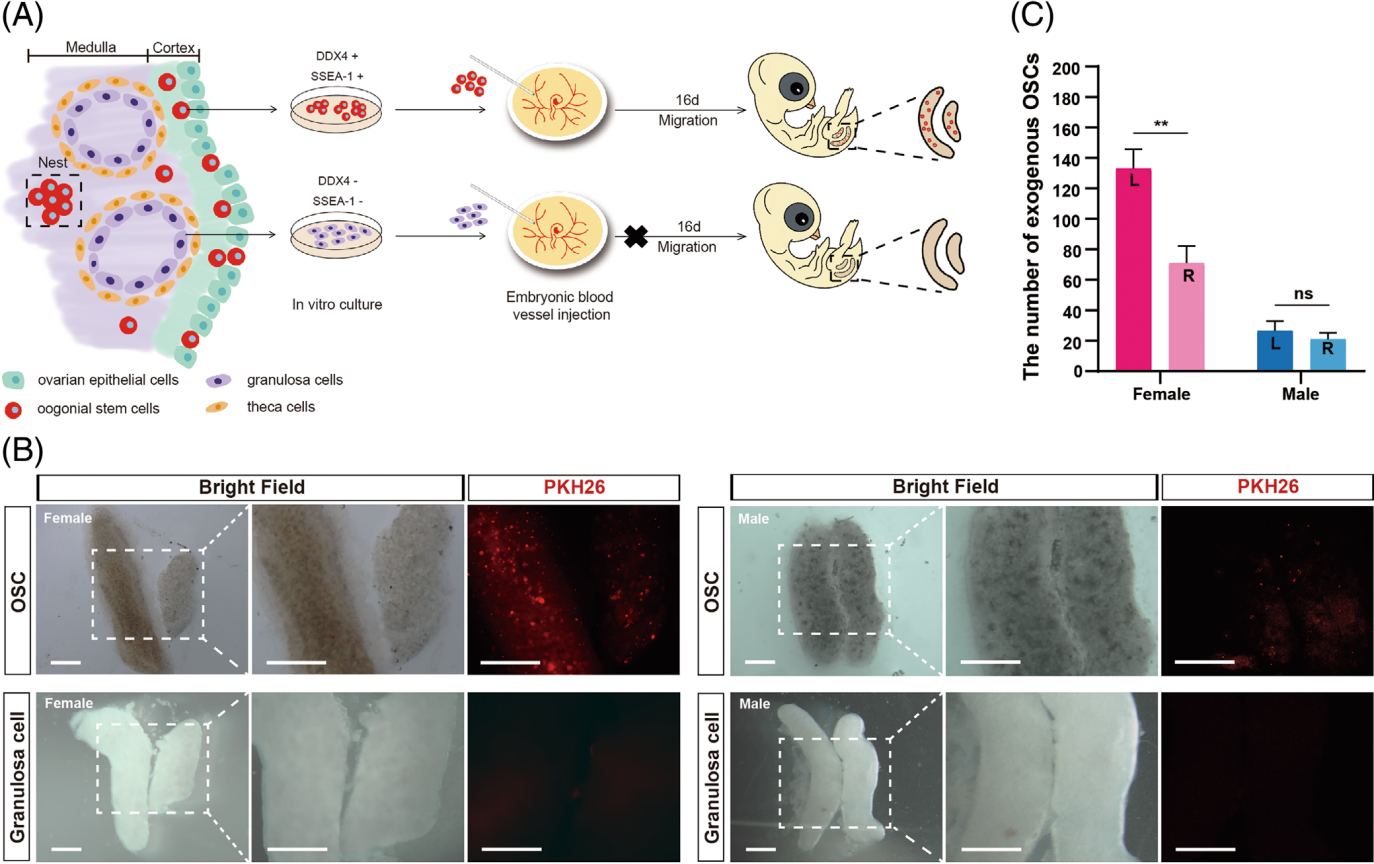
The transplantation and migration of chicken oogonial stem cells (OSCs). (A) Cell transplantation assay to assess the migration and differentiation potential of the cultured OSCs. (B) Representative pictures show that the PKH26‐labelled OSCs colonized the developing gonads, whereas the PKH26‐labelled granulosa cells could not colonize the developing gonads. Scale bar = 0.5 mm. (C) The number of labelled OSCs located in the female left gonad was significantly higher than that in the right gonad. The OSCs also migrated into male gonads, but they were equally distributed in the left and right gonads. Data are the mean ± SEM (*n* = 5), **p < 0.01. ns, not significant by Student's *t*‐test.

**FIGURE 5:**
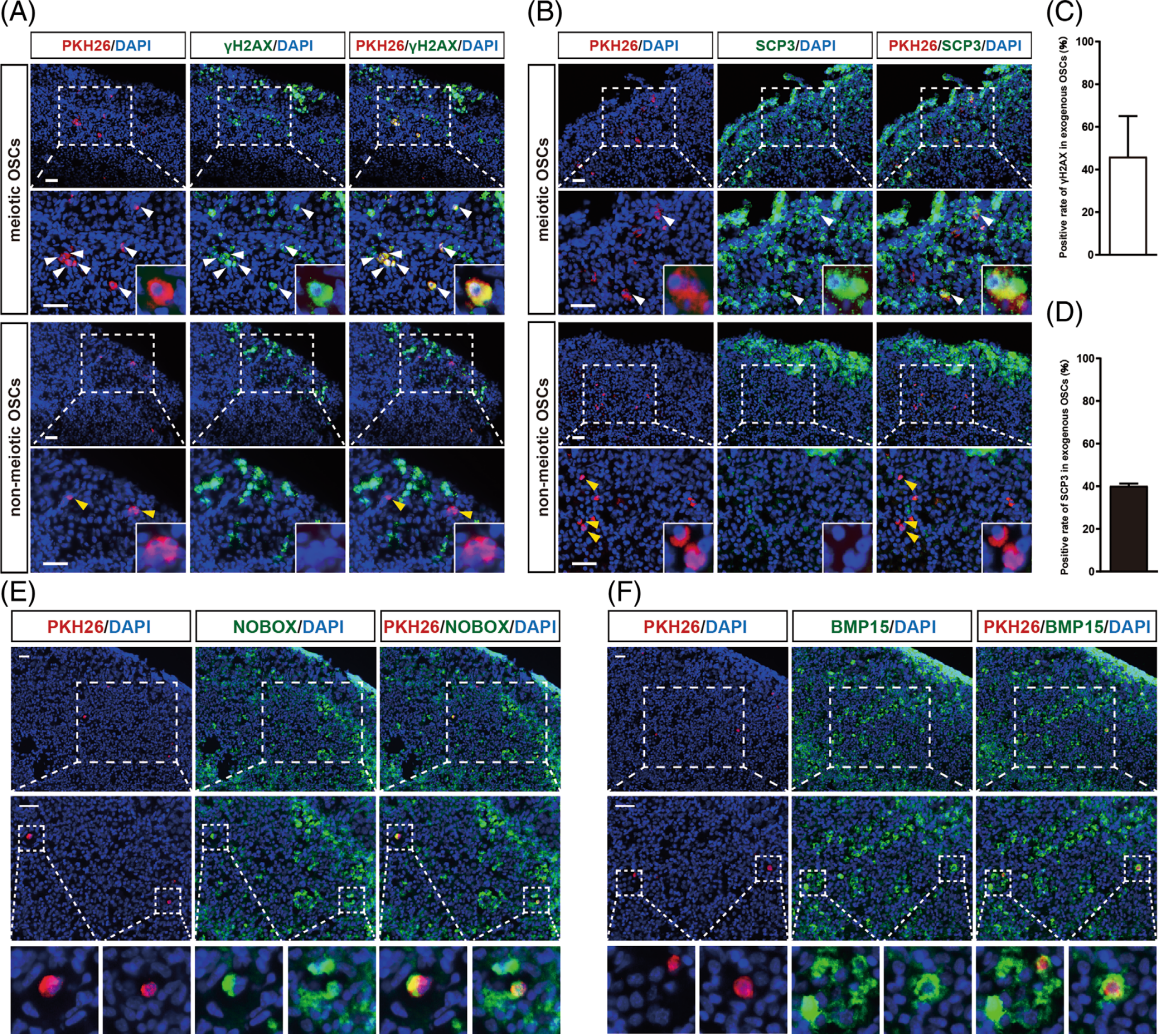
Verification of differentiation ability of chicken oogonial stem cells (OSCs). (A) The distribution of transplanted OSCs (PKH26) and meiotic cells (γH2AX) in female embryonic gonads. The area in the dashed box is magnified in the bottom panels. Note that some labelled OSCs were positive for γH2AX (white arrowheads), while others were negative for γH2AX (yellow arrowheads). Scale bar = 50 μm. (B) Colocalization of PKH26 and SCP3 in female embryonic gonads. The area in the dashed box is magnified in the bottom panels. Note that some labelled OSCs were positive for SCP3 (white arrowheads), while others were negative for SCP3 (yellow arrowheads). Scale bar = 50 μm. (C) The proportion of meiotic OSCs as quantified by γH2AX staining. (D) The proportion of meiotic OSCs as quantified by SCP3 staining. Data are the mean ± SEM (*n* = 5). (E) Colocalization of PKH26 and NOBOX in postnatal chicken ovaries. Some PKH26 labelled OSCs were observed in germ cell nests and individual primordial follicles, which were labelled with NOBOX (dashed box). The area indicated by the dashed box in the overview is magnified at the bottom. Scale bar = 50 μm. (F) Colocalization of PKH26 and BMP15 in postnatal chicken ovaries. Some PKH26 labelled OSCs were observed in germ cell nests and individual primordial follicles, which were indicated by BMP15 (dashed box). The area indicated by the dashed box in the overview is magnified at the bottom. Scale bar = 50 μm.

During embryonic development, chicken germ cells adopt a unique migration pattern in which they circulate within the bloodstream to reach the gonads.^18^ To determine the migration and differentiation capabilities of the OSCs, we transplanted PKH26‐labelled OSCs into the blood vessels of the developing embryos (E2.5) (Figure 4A). The same number of GCs was also injected as a control. Ten days later, we collected the embryos (E12.5) and identified PKH26‐positive cells in the gonads. Interestingly, the OSCs could colonize both female and male gonads (Figure 4B,C), indicating that adult OSCs might even have sex differentiation bipotential, equivalent to PGCs.^34^Although the same number of OSCs were injected into the blood of individuals of both sexes, the OSCs had an obvious preference for female gonads (Figure 4B). We also observed a significantly higher number of OSCs distributed in the left developing gonad than in the right regressing gonad in females (Figure 4C). In contrast, no labelled GCs were found in any of the gonads of either sex (Figure 4B). After confirming the migration capacity of the OSCs, we examined whether they can differentiate into oocytes. In the E18.5 female left gonads, we observed that numerous PKH26‐labelled OSCs were positive for γH2AX or SCP3 (Figure 5A,B), indicating that the transplanted OSCs could initiate meiosis. However, only 40% of OSCs could enter meiosis, and the proportion of meiotic OSCs was much lower than that of endogenous germ cells in the original ovaries (>80%) (Figure 5C,D; Figure 2C,D). Therefore, the cultured OSCs could efficiently migrate into the gonad, but some of them lost the ability to initiate meiosis.

Next, to further evaluate the fate of the labelled OSCs postnatally, we collected the ovaries from the post‐hatching animals and examined the distribution of labelled OSCs. The oocyte‐specific markers BMP15^35^ and NOBOX^36^ were employed to identify the oocytes. In the postnatal ovaries at 4 days after hatching, during which time the germ cell nests were frequently breakdown into individual oocytes to assemble the primordial follicles.^37^ We found that the PKH26‐labelled OSCs were located within the nests as well as the derived primordial follicles (Figure 5E,F). Hence, we confirmed that the OSCs can actively contribute to the oogenesis in postnatal ovaries.

## 4 DISCUSSION

It has long been accepted that in mammals, all oogonia enter meiosis during fetal life, with no remaining germ stem cells left in the ovary, in contrast to spermatogonia, which are present in the testis throughout life.^38^, ^39^ Nevertheless, the continuation of oogenesis into adulthood has been well documented in non‐mammalian species, including flies,^40^ fishes,^14^ amphibians^15^ and reptiles.^16^ This study provided the first evidence of the existence and functional relevance of OSCs in birds, which are considered to have undergone convergent evolution with mammals with respect to many physiological features.^41^ Thus, it seems that the active formation of oocytes and follicles in the ovaries of adult females during the reproductive period is a basic biological process that has been conserved through the evolution of the animal kingdom toward mammals.

In lower vertebrates such as fish and amphibians, an adult female is capable of laying hundreds of eggs at one time, since ovarian OSCs can maintain efficient proliferation and execute meiosis to regularly generate a massive number of mature oocytes.^42^ In contrast, higher vertebrates such as mammals produce a very limited number of mature eggs and the derived offspring throughout life, and the contribution from de novo oogenesis during adulthood was also evidenced by genetic fate mapping and targeted gene disruption or cell ablation studies.^43^, ^44^ Birds and mammals are the only warm‐blooded animals and share many similarities in ovarian physiology and reproductive biology. Both birds and mammals usually produce one or a few eggs at one time to ensure the health and survival of their offspring. While the occurrence of de novo oocyte formation was still under debate in adult mammals, there is no available information related to adult oogenesis in any bird species.

This study confirmed the presence of OSCs in the birds, but the origin of these cells in ovaries is still unknown. Although other putative germ cells originating from bone marrow^45^ or the very small embryonic‐like stem cells that reside in the ovary^46^ may give rise to eggs in the adult ovary, their developmental lineages are unclear. It is natural to assume that OSCs could be derived from PGCs in the developing gonads. In accordance with this hypothesized cellular ontogeny, our analysis of the female gonads showed that not all PGCs underwent meiosis during the meiotic wave. These findings raised the possibility that a small portion of PGCs escaped embryonic meiosis stimulation and maintained the capacity to restart meiosis later on. These cells can continue to perform mitosis or remain quiescent to survive in the developing ovary, waiting for the appropriate stimulation to initiate mitosis in adulthood. The behaviour of the meiotic germ cells identified in the adult chicken ovary is in agreement with this assumption. These potential OSCs were isolated, and their capability for mitosis and meiosis were verified both in vitro and in vivo. Therefore, analogous to de novo oocyte formation in lower vertebrates, chickens also retain functional OSCs in adult ovaries.

What possible advantage would be gained from the maintenance of this pool of OSCs in chickens? A recent study suggested that once the follicle pool is exhausted or below a specific threshold, the birds would theoretically enter reproductive senescence.^47^ This finding indicated that maintaining a balance in the number of available primordial follicles is necessary for maintaining normal laying.^48^ Thus, a small proportion of OSCs could serve as the cellular source for the addition of new primordial follicles during laying. This is also the case in mammals that specific ablation of differentiating OSCs in adult ovaries resulted in reduced oocyte pool.^44^ However, to fully exploit the potential of OSCs to support and enhance the active production of eggs, further study is needed to uncover the molecular mechanisms that control the quiescence and activation of these cells.

In avian species, cryo‐preservation of oocytes and embryos is not possible due to their large quantities of deposited lipids.^49^, ^50^ The OSCs in adult avian ovaries offer an alternative material for the conservation of bird species, particularly for some local and extant populations with valuable genetic resources. In addition, OSCs isolated from deceased or infertile individuals of endangered wild bird species could be used to produce donor‐derived eggs by host hens, significantly impacting future avian conservation. This study also verified that chicken OSCs can endure long‐term cryopreservation and tolerate freezing–thawing cycles, allowing the efficient maintenance, transportation, and utilization of the germplasm of interest.

In conclusion, the identification of functional OSCs within adult avian ovaries opens up new opportunities for the conservation of bird species through the rescue and maintenance of valuable genetic diversity as well as the improvement of egg production. Further work is required to understand what signals control the mitotic and meiotic activity of chicken OSCs to shed light on the basic and clinical applications of mammalian OSCs.

## AUTHOR CONTRIBUTIONS

*Conceived and designed the experiments*: Lu Meng and Guiyu Zhu. *Performed the experiments*: Lu Meng, Yun Zhang and Yuxiao Ma. *Analysed the data*: Yao Hua, Lu Meng, Heng Wang, Xianyao Li and Yunliang Jiang. *Wrote and reviewed the article*: Lu Meng and Guiyu Zhu.

## CONFLICT OF INTEREST

The author declares that there is no conflict of interest that could be perceived as prejudicing the impartiality of the research reported.

## Supporting information


**FIGURE S1.** The characterization of freshly isolated oogonial stem cells (OSCs). (A) The immunofluorescence staining for DDX4 and stage‐specific embryonic antigen‐1 (SSEA‐1) in freshly isolated cells. Chicken OSCs and primordial germ cells (PGCs) were purified by SSEA‐1 based magnetic‐assisted cell sorting. The dashed box in the overview is magnified in the insert. Scale bars = 20 μm. (B) Percentage of DDX4 and SSEA‐1 double‐positive cells in freshly isolated cells to show the purity of the OSCs or PGCs. Data are the mean ± SEM, *n* = 5, not significant by Student's *t‐*test.
**TABLE S1.** Primers for qRT‐PCR

## Data Availability

The data are available from the corresponding author upon reasonable request.
