# Supplementary material for: Identification of oogonial stem cells in chicken ovary
Source: Cell Prolif. 2022 Dec 16;56(3):e13371. doi: 10.1111/cpr.13371 (PMC9977656; doi:10.1111/cpr.13371)
Supplement: Supplementary file 1 — FIGURE S1. The characterization of freshly isolated oogonial stem cells (OSCs). (A) The immunofluorescence staining for DDX4 and stage‐specific embryonic antigen‐1 (SSEA‐1) in freshly isolated cells. Chicken OSCs and primordial germ cells (PGCs) were purified by SSEA‐1 based magnetic‐assisted cell sorting. The dashed box in the overview is magnified in the insert. Scale bars = 20 μm. (B) Percentage of DDX4 and SSEA‐1 double‐positive cells in freshly isolated cells to show the purity of the OSCs or PGCs. Data are the mean ± SEM, n = 5, not significant by Student's t‐test. TABLE S1. Primers for qRT‐PCR [file CPR-56-e13371-s001.docx]

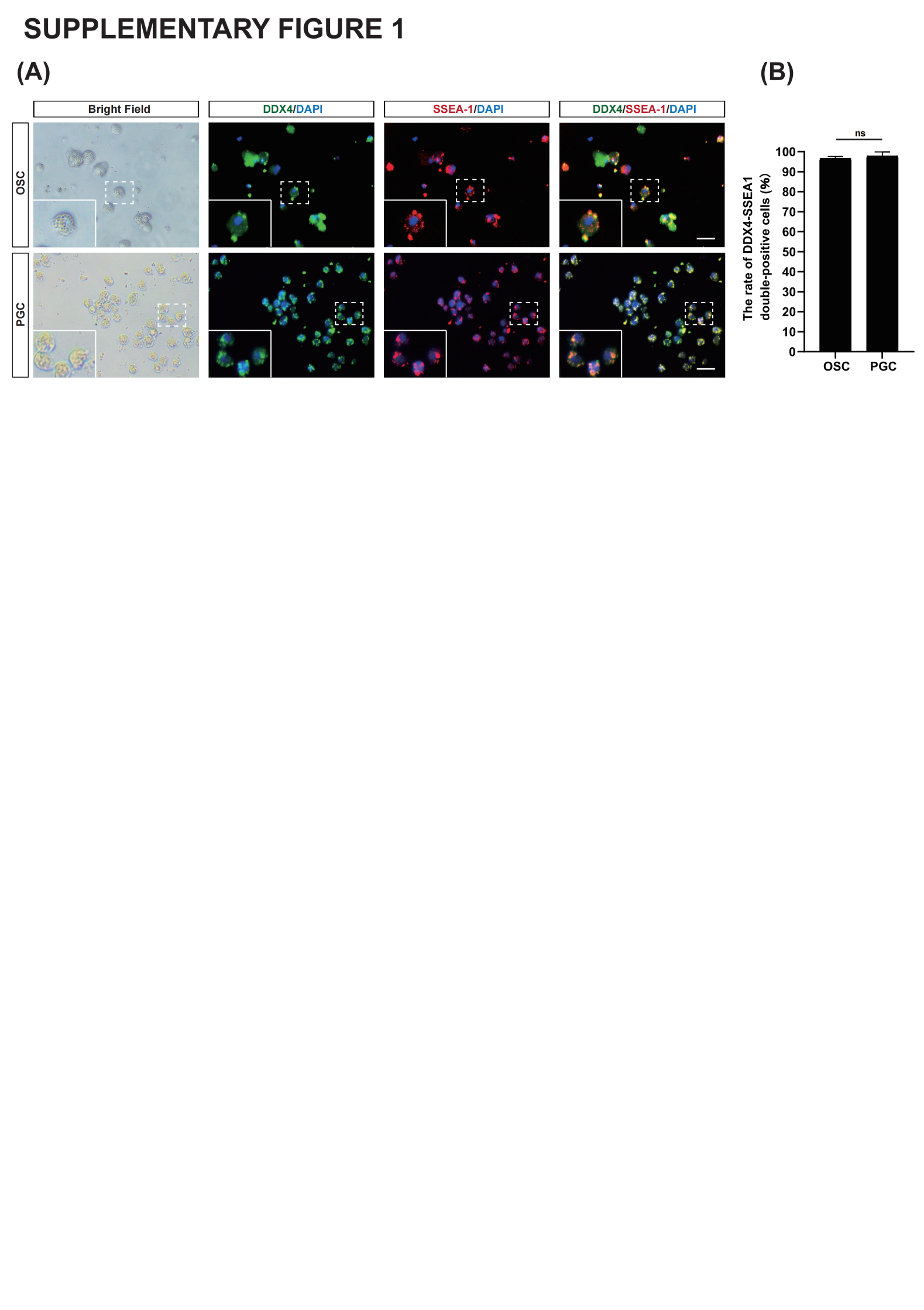


**Supplementary Figure 1. The characterization of freshly isolated OSCs.**

1. The immunofluorescence staining for DDX4 and SSEA-1 in freshly isolated cells. Chicken oogonial stem cells (OSCs) and primordial germ cells (PGCs) were purified by SSEA-1 based MACS. The dashed box in the overview is magnified in the insert. Scale bars = 20 μm.
2. Percentage of DDX4 and SSEA-1 double-positive cells in freshly isolated cells to show the purity of the OSCs or PGCs. Data are the mean ± SEM, n=5, not significant by Student’s t test.

**Supplementary Table 1. Primers for qRT-PCR**

| **Gene** | **Sense primer** | **Anti-sense primer** |
| --- | --- | --- |
| **SCP3** | CTGTATTTCAGCAGTGGGATG | TGCGAAGTTCATTTTGTGC |
| **STRA8** | GTGAGGGACAGTGGAGGTAA | CAGAAATGCCGCTTGTAAAT |
| **SPO11** | TGGTCAGAAAGCTGTGGGATA | TGATGTCGGATGGAAGGAGA |
| **DMC1** | AGATGACAACAAGACGAGCACT | CAATCCCACCACCCAGAA |
| **DDX4** | AGGCGTGGATGGCTAACTCT | GGACGTACAACAGACTCTCTGC |
| **SSEA-3** | GAGGCGCTACAACATCACGG | AGGGCGACTCGAAGTTCATC |
| **OCT4** | CTCAATGAGGCAGAGAACACG | CAGACCCGGACAACGTCTTT |
| **SOX2** | AAACCAAGACCCTGATGAAGA | ATCCCATAGCCTCCGTTG |
| **NANOG** | AGCAGACCTCTCCTTGACCA | TTCCTTGTCCCACTCTCACC |
| **C-KIT** | GCATCCAGCAATGGTGAC | AAGTTGCGTTGGGTCTAT |
| **C-MYC** | ATCGACCCCTCGGTGGTCTT | GCAGCGTAGTTGTGTTGGTG |
| **SALL4** | GTCCACTGCGGACCCCAACG | GGTGGAGAAGGCACGGCCAC |
| **PRDM1** | AAGAATCTGGTGAAAGGGAG | GCAGTTTGATGCGTATTTG |
| **DAZL** | GCACCGCAATTCCATAGCGT | CTGTGGTGGAGCCTGATAGT |
| **FOXL2** | TGTCCGGGATCTACCAGTAC | CTCGAACATGTCCTCGCA |
| **AMH** | GTGGGGATGCAAAAACCGTG | AAAGGCTCTGCTCCTGTGTC |
| **CYP19A1** | TGTTCCATCACGCTATTT | GATTCTTGTTTGGGCTTC |
| **STAR** | CCCTGCAGAAATCACTCAGC | AGAGTGTCCTTCCCAACCCT |
| **INSL3** | GATGAGGCGGATACGTGCTTG | GAGATCCTGTTGTGGCTGAGGAA |
| **CYP17A1** | GAAGTTCCAGCGCAAACTGG | GGAGCAGACCACATTGGTGA |
| **GAPDH** | CCTCTCTGGCAAAGTCCAAG | CATCTGCCCATTTGATGTTG |
